# Definition of Erythroid Differentiation Subsets in Normal Human Bone Marrow Using FlowSOM Unsupervised Cluster Analysis of Flow Cytometry Data

**DOI:** 10.1097/HS9.0000000000000512

**Published:** 2020-12-21

**Authors:** Marie C. Béné, Olof Axler, Despoina Violidaki, Francis Lacombe, Mats Ehinger, Anna Porwit

**Affiliations:** 1 Hematology Biology, Nantes University Hospital, Nantes, France; 2 Department of Clinical Sciences, Oncology and Pathology, Lund University, Faculty of Medicine, Lund, Sweden; 3 Hematology Biology, Bordeaux University Hospital Haut Leveque, Bordeaux, France.

Erythropoiesis is a complex process that ensures the production of about 200 billion erythrocytes every day, at a rate of almost 2 million per second in humans. Knowledge of the maturation process of red blood cells comes mostly from morphological analysis of bone marrow (BM) smears or biopsies, colony unit formation studies in cultures, and electron microscopy.^1-3^ Multiparameter flow cytometry (MFC) explorations added new information about the kinetics of expression and the role of various erythroid lineage-associated markers such as CD105 (endoglin), CD71 (transferrin receptor), and CD36 (thrombospondin receptor).^4,5^ A relatively limited number of MFC studies have been published concerning erythroid maturation,^6,7^ lately aiming at providing information about erythroid lineage alterations in early stages of unilineage myelodysplastic syndromes characterized by isolated anemia.^8^ The Red score, proposed in 2013, presented a bold approach using nonlysed BM samples in a panel where the addition of a DNA dye allowed to exclude from analysis mature red blood cells that have lost their nucleus.^9^ More recently, our group in Lund published a similar MFC approach, including multiparametric radar representation, to assess erythroid maturation with a single 7-color panel.^10^ This strategy allowed to delineate and confirm the differentiation pattern of erythropoiesis characterized by the following steps: (1) loss of CD117, (2) transient expression of CD105, and (3) appearance and decrease of CD36 and CD71. This highly reproducible representation, however, relied on traditional supervised sequential gating along the continuum of maturation.

Unsupervised analysis of cytomics data is an emergent and growing field mostly applied to mass cytometry but applicable to classical fluorescence-based MFC.^11^ Among the various software developed, the R-based (Bioconductor) Flow SOM (for flow-self organizing maps) solution was recognized as both robust and fast.^12,13^ This strategy has been applied by our group to the definition of normal hematopoiesis using 4 different antibody combinations. Merged list-mode files of 16 (for lymphoid markers) or 19 (for myeloid markers) normal BM samples were used to define reference patterns.^14,15^ All samples had been processed in the same harmonized^16^ lysis-no wash fashion. FlowSOM self-delineated 100 cell subset “nodes” in each of 4 so-called minimal spanning trees (MSTs). By coupling FlowSOM unsupervised segregation of these cell subsets and the versatile Kaluza software, a rapid identification of the size (number of cells) and immunophenotypic features of each node is easily done. Up to 24 different hematopoietic subsets (comprising different nodes) could be identified, including progenitors, granulocytic, monocytic, and lymphoid maturation.^14,15^

Here, we applied the same strategy to the list-mode files of 11 unlysed normal BM samples stained with our published so-called ERY (erythroid) tube.^10^ All 11 BM samples had normal erythropoiesis by cytomorphology and all individuals had normal complete blood counts (Supplemental Table 1, http://links.lww.com/HS/A120). Of note, the size of the panel used is limited by the broad spectrum of emission fluorescence of emission fluorescence of the Draq5 stain, used here primarily to set the acquisition gate on nucleated cells and then, during analysis, to define proliferating cell clusters. The files were subjected to compensation checking and fluorescence was normalized using lymphocytes as a reference as described in detail previously.^15^ Then, the normalized files were merged with the Kaluza merging tool. The resulting single merged file was submitted to unsupervised analysis by FlowSOM with the set-seed option^15^ allowing the software to generate 24 MST of 100 nodes. The nodes belonging to the erythroid lineage were located on the MST through back gating and coloring of a classical CD45/side scatter dot plot.^15^ The graphical MST providing the best grouping, yet clear separation of the nodes of interest, was then chosen (Figure 1). Each node was individually studied in Kaluza, recording the number of events and the mean fluorescence intensity (MFI) of each marker. The frequency of cells in each node was then calculated both as a fraction of the whole sample and of the erythroid population (Figure 2 and Table 1).

**Table 1:** Subsets of Erythropoietic Cells Identified in a Merged File of 11 Normal Bone Marrows.

Subset	% Cells*	CD117		CD105		% Draq5^high^†		DraqMFI		CD36		CD71		CD45		FSC		SSC	
		Mean	Median	Mean	Median	Mean	Median	Mean	Median	Mean	Median	Mean	Median	Mean	Median	Mean	Median	Mean	Median
EARLY DIV	1.0/06.4	2.72	2.82	2.63	2.36	74.00	67	0.13	0.12	4.94	5.08	4.80	4.78	0.40	0.41	0.68	0.67	0.59	0.59
INT DIV	2.9/15.1	1.08	1.07	1.86	2.00	54.67	78.50	0.12	0.13	4.19	4.33	3.88	4.16	0.34	0.34	0.63	0.66	0.58	0.59
LATE DIV	4.2/20.2	1.27	1.23	1.34	1.37	97.25	99.00	0.14	0.15	4.65	4.68	4.46	4.46	0.35	0.35	0.65	0.64	0.59	0.59
HIGH 36/71	6.6/20.7	1.16	1.08	1.12	1.10	8.75	8.25	0.10	0.10	4.15	4.17	4.93	4.93	0.34	0.34	0.62	0.61	0.60	0.56
INT 36/71	7.5/28.4	1.09	0.97	1.17	1.13	3.36	3.00	0.09	0.10	4.00	4.02	3.54	3.56	0.33	0.33	0.59	0.58	0.56	0.55
Low DRAQ5	0.9/09.1	1.05	1.05	1.05	1.05	0.00	0.00	0.05	0.05	3.47	3.47	3.61	3.61	0.32	0.32	0.58	0.58	0.55	0.55

**BM = bone marrow, FSC = forward scatter, INT = intermediate, SSC = side scatter.**

*In total BM/within erythropoiesis.

†% Draq5^high^ positive events were considered the fraction of dividing cells.

**Figure 1.**
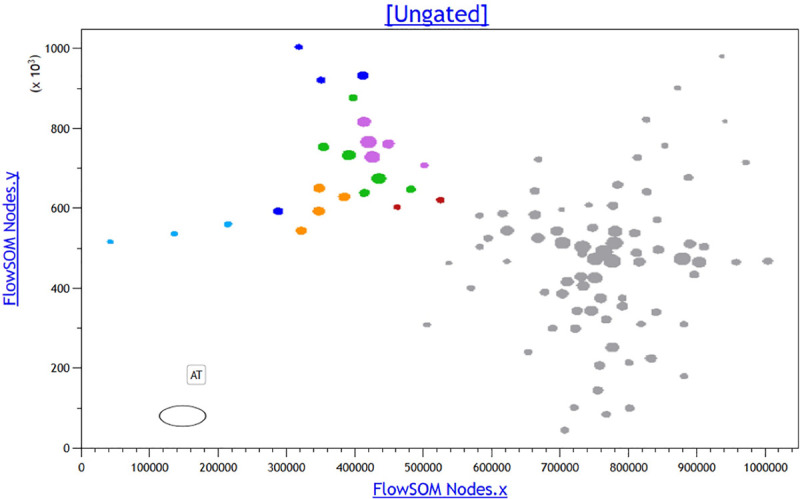
**FlowSOM representation of the merged file of 11 normal BMs (selected minimal spanning tree).** Nodes belonging to erythropoiesis were characterized (Table 1) and colored as follows: EARLY DIV sky blue, INT DIV navy blue, LATE DIV amber, HIGH 36/HIGH 71 green, INT 36/INT 71 violet, and DRAQ LOW crimson. Nodes belonging to other cell subsets in the BM are colored in gray. The size of the individual nodes reflects the number of events pertaining to each node. **BM = bone marrow, INT = intermediate.**

**Figure 2.**
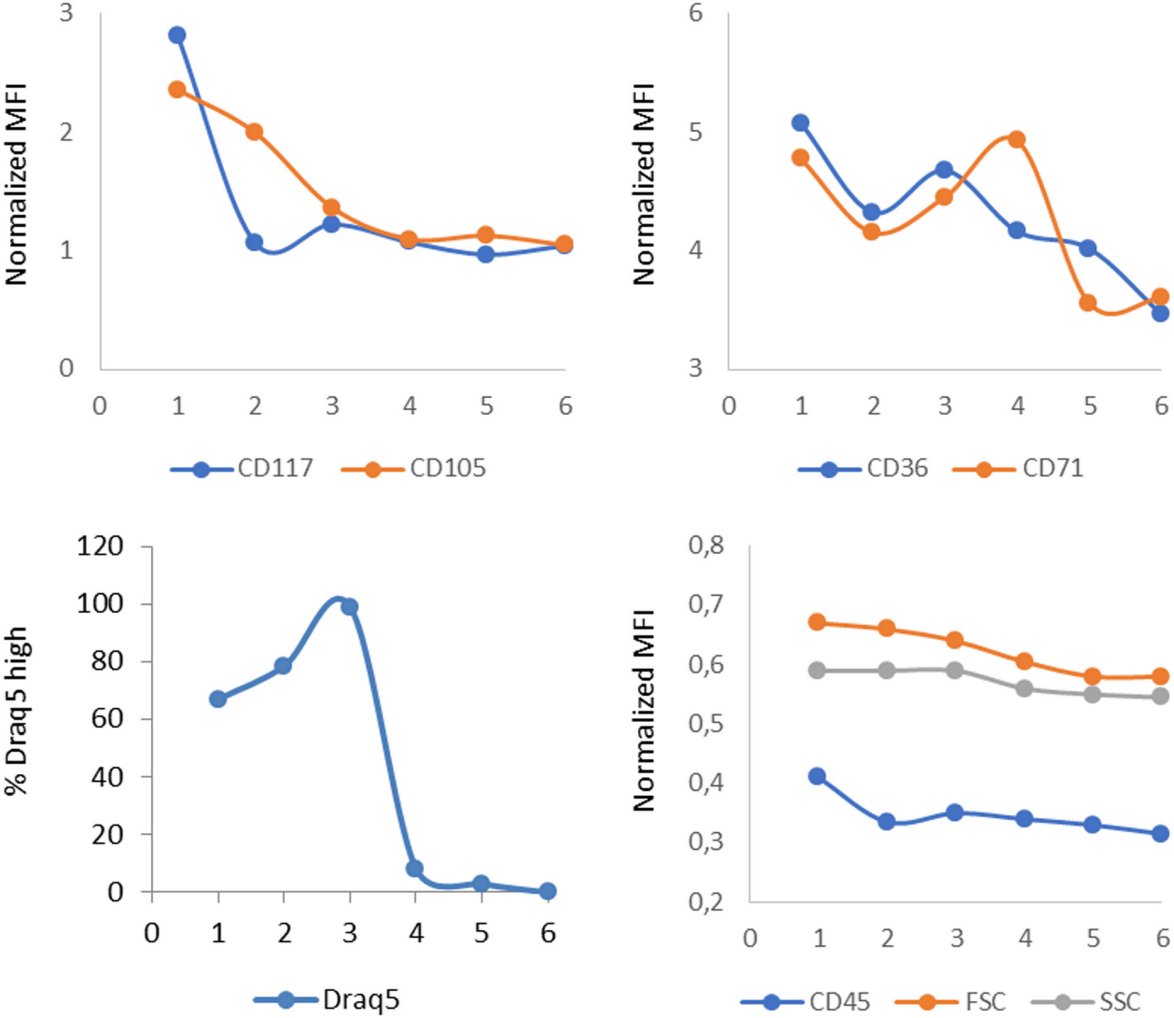
**Kinetic expression of erythroid markers in the 6 identified subsets of erythropoiesis (Table 1 and Figure 1). FSC = forward scatter, MFI = mean fluorescence intensity, SSC = side scatter.**

In the chosen MST, 24 nodes qualified as belonging to various known stages of erythroid differentiation, built on the expression of CD45, CD117, CD105, CD36, and/or CD71. In turn, based on fluorescence intensity of each of these markers, these 24 nodes could be classified into 6 subsets of erythropoietic maturation (Figures 1 and 2 and Table 1). These subsets refined the known sequence of marker expression within erythroid maturation. The cutoffs for HIGH versus intermediate (INT) CD36 and CD71 normalized mean fluorescence intensity (MFI) were 4.1 and 4.59, respectively. The lowest normalized MFI for positive expression of CD36 and CD71 in normal BM were 3.83 versus 2.52, respectively. The cutoff for high Draq5 normalized MFI was 0.1, while very low Draq5 was between 0.01 and 0.06. Of interest, MFI of the DNA dye Draq5, which parallels DNA content, could be used more precisely than in our Radar approach^10^ to identify proliferative subsets and correlate this with their immunophenotype. Three subsets of dividing (DIV) cells (respectively dubbed EARLY DIV, INT DIV, and LATE DIV) were identified by their high percentage of Draq5^hi^ cells (Table 1). They differed by the presence of CD117 (EARLY DIV), then by CD105 expression in the absence of CD117 (INT DIV), and finally by a CD117^–^/CD105^–^ immunophenotype (LATE DIV). The normalized MFI of CD36 and CD71 was high in these populations as defined by the cutoffs. The next 2 subsets (dubbed HIGH 36/71 and INT 36/71) were consistently negative for CD117 and CD105. They also had a low fraction of Draq5^hi^ cells suggesting that they nearly stopped dividing. The last small identified subset had very low Draq5 content (LOW DRAQ5) and could correspond to apoptotic cells or cells having just expelled their nucleus (reticulocytes).^17^

The EARLY DIV population, corresponding to the earliest erythroid progenitors (CD117+), had the highest CD45 expression and cell size measured by forward scatter. CD45 MFI was progressively lower in later stages of erythropoietic differentiation. Forward scatter was also progressively lower while side scatter remained similar in most subsets.

In conclusion, we report here on the use of unsupervised MFC analysis of normal BM, with an erythroid-specific panel and the no-lysis strategy, to delineate discrete subsets of erythroid maturation. Although the 24 nodes allocated to erythropoiesis could be ultimately grouped in 6 major populations, subtle differences, significant enough for FlowSOM to single them out, existed between each node within the erythroid compartment. These differences were not obvious enough to define the subsets by supervised analysis. Because eleven normal BM samples were used to obtain a representative pattern, differences between nodes related to individual variations were minimized. Thus, the robustness of FlowSOM (for unsupervised analysis) and of the panel used^10^ provide good confidence about this reference pattern. Of course, different panels using other erythroid-specific markers such as CD123, E-cadherin, EPO-R, or coxsackie adenovirus receptor could provide further information in a similar analytical approach. As previously shown with the radar approach,^10^ this new strategy is likely to provide important information for the analysis of diseases involving the erythroid lineage such as myelodysplastic syndrome, renal failure, vitamin B or iron deficiency, and other conditions. This is a currently ongoing investigation.

## Supplementary Material


