# Supplementary material for: Definition of Erythroid Differentiation Subsets in Normal Human Bone Marrow Using FlowSOM Unsupervised Cluster Analysis of Flow Cytometry Data
Source: Hemasphere. 2020 Dec 21;5(1):e512. doi: 10.1097/HS9.0000000000000512 (PMC7755522; doi:10.1097/HS9.0000000000000512)

	Leukopenia	Normal	Leukocytosis	P value	Thrombocyto-penia	Normal	Thrombocytosis	P value
**Number**	7895	131364	3932		2312	139239	1640	
**Male sex (%)**	38.4	41.9	37.5	<0.001	68.6	41.5	13.4	<0.001
**Age (years)**	46.4 ± 12.4	44.3 ± 12.8	42.8 ± 11.3	<0.001	47.5 ± 14.4	44.4 ± 12.7	43.5 ± 11.7	<0.001
**BMI (kg/m^2)**	24.4 ± 3.4	26.1 ± 4.3	27.2 ± 5.3	<0.001	25.9 ± 4.0	26.0 ± 4.3	26.8 ± 5.2	<0.001
**Smoking**				<0.001				<0.001
Current/former/never	6.9/38.5/54.6	20.3/33.1/46.6	68.2/13.3/18.5		17.8/36.4/45.8	20.9/32.8/46.3	28.2/30.0/41.8	
**Number of medications used**	0 (0 – 1)	1 (0 – 2)	1 (0 – 3)	<0.001	0 (0 – 2)	1 (0 – 2)	1 (0 – 2)	<0.001
**Alcohol use**				<0.001				<0.001
Non/light/moderate/heavy	19.6/51.9/21.7/6.8	21.3/50.2/20.4/8.1	28.3/43.0/18.0/3.6		19.2/48.4/21.4/2.2	21.3/50.2/20.4/8.1	35.2/46.5/13.2/5.2	
**Anemia (%)**	9.0	3.9	2.6	<0.001	4.5	4.0	18.3	<0.001
**MCV (fL)**	89.9 ± 4.5	89.8 ± 4.1	91.2 ± 4.6	<0.001	89.6 ± 4.5	89.9 ± 4.1	88.3 ± 6.7	<0.001

	Neutropenia	Normal	Neutrophilia	P value	Lymphopenia	Normal	Lymphocytosis	P value
**Number**	6756	13214	2020		473	134530	5887	
**Male sex (%)**	37.4	41.8	34.5	<0.001	48.4	41.8	34.8	<0.001
**Age (years)**	45.4 ± 13.8	44.4 ± 12.7	42.6 ± 10.6	<0.001	47.8 ± 12.0	44.5 ± 12.7	41.3 ± 14.0	<0.001
**BMI (kg/m^2)**	24.3 ± 3.4	26.1 ± 4.3	26.7 ± 5.3	<0.001	25.1 ± 3.7	26.0 ± 4.3	26.8 ± 5.1	<0.001
**Smoking**				<0.001				<0.001
Current/former/never	8.8/37.3/53.9	20.5/33.1/46.5	60.8/15.1/24.1		11.8/36.7/51.5	19.3/33.6/47.2	49.2/20.2/30.6	
**Number of medications used**	0 (0 – 1)	1 (0 – 2)	1 (0 – 2)	<0.001	0 (0 – 3)	1 (0 – 2)	1 (0 – 2)	<0.001
**Alcohol use**				<0.001				<0.001
Non/light/moderate/heavy	17.3/52.6/22.5/7.6	21.5/50.1/20.3/8.1	27.7/45.7/17.6/9.0		21.6/50.7/19.7/8.1	21.3/50.3/20.4/8.0	23.0/48.3/19.0/9.6	

**Anemia (%)**	7.5	4.0	3.0	<0.001	9.1	4.3	2.3	<0.001
**MCV (fL)**	90.0 ± 4.3	89.8 ± 4.2	91.3 ± 4.6	<0.001	90.1 ± 4.9	89.8 ± 4.2	90.6 ± 4.2	<0.001
	**Monocyto-penia**	**Normal**	**Monocytosis**	**P value**		**Normal**	**Basophilia**	**P value**
**Number**	9713	129056	2121			**140877**	13	
**Male sex (%)**	25.9	42.3	62.5	<0.001		41.5	46.2	0.734
**Age (years)**	43.4 ± 11.8	44.5 ± 12.8	45.4 ± 13.4	<0.001		44.4 ± 12.7	52.4 ± 8.2	0.024
**BMI (kg/m^2)**	24.8 ± 3.8	26.1 ± 4.3	26.8 ± 4.8	<0.001		26.0 ± 4.3	26.3 ± 7.1	0.567
**Smoking**				<0.001				0.066
Current/former/never	9.7/36.4/53.9	20.8/32.9/46.3	54.3/22.1/23.6			20.5/33.0/46.5	38.5/46.2/15.4	
**Number of medications used**	1 (0 – 1)	1 (0 – 2)	1 (0 – 2)	<0.001		1 (0 – 2)	1 (0 – 2)	0.978
**Alcohol use**				<0.001				0.675
Non/light/moderate/heavy	22.9/54.4/18.0/4.7	21.3/50.0/20.5/8.2	21.3/40.9/22.4/2.7			21.4/50.2 20.4/8.1	16.7/41.7/25.0/16.7	
**Anemia (%)**	6.4	2.0	2.9	<0.001		4.1	7.7	0.520
**MCV (fL)**	89.6 ± 4.2	89.8 ± 4.2	91.2 ± 4.4	<0.001		89.8 ± 4.2	90.5 ± 8.7	0.843
		**Normal**	**Eosinophilia**	**P value**	**1st quintile**	**2nd-4th quintile**	**5th quintile**	**P value**
**Number**		133329	7561		28201	84714	27975	
**Male sex (%)**		41.0	49.8	<0.001	40.4	42.7	39.0	<0.001
**Age (years)**		44.4 ± 12.7	44.0 ± 13.3	0.007	43.3 ± 14.4	44.4 ± 12.5	45.4 ± 11.7	<0.001
**BMI (kg/m^2)**		26.0 ± 4.3	26.0 ± 4.3	0.545	25.3 ± 3.9	26.1 ± 4.3	26.4 ± 4.7	<0.001
**Smoking**								
Current/former/never		9.9/33.2/46.9	30.9/29.1/40.1	<0.001	18.3/34.0/47.7	20.4/33.0/46.6	22.9/32.0/45.1	<0.001
**Number of medications used**		1 (0 – 2)	1 (0 – 2)	<0.001	1 (0 – 2)	1 (0 – 2)	1 (0 – 2)	<0.001
**Alcohol use**				<0.001				<0.001
Non/light/		21.4/50.4/	20.5/46.6/		17.8/51.4/	21.3/50.2/	25.1/49.0/	

moderate/heavy	20.2/7.9	22.6/10.3		21.6/8.8	20.4/8.0	18.5/7.4	
**Anemia (%)**	3.9	0.2	0.005	3.8	4.0	5.0	<0.001
**MCV (fL)**	89.8 ± 4.2	90.1 ± 4.2	<0.001	90.0 ± 4.0	89.8 ± 4.1	89.9 ± 4.5	<0.001

SDC, Table 1. Baseline characteristics for participants with and without peripheral blood cell count abnormalities. Data are given as mean ± SD, median (IQR) when not normally distributed, or percentage. BMI, body mass index; IQR, interquartile range; MCV, mean corpuscular volume; SD, standard deviation.

	Leukopenia		WBC in normal range		Leukocytosis	
	Mean ± SD	Median (IQR)	Mean ± SD	Median (IQR)	Mean ± SD	Median (IQR)
**Physical functioning**	92.0 ± 13.0	95.0 (90.0-100.0)	90.6 ± 14.2	95.0 (90.0-100.0)	85.2 ± 19.6	90.0 (80.0-100.0)
**Social functioning**	88.5 ± 17.3	100.0 (87.5-100.0)	87.7 ± 18.0	100.0 (75.0-100.0)	83.0 ± 21.7	87.5 (75.0-100.0)
**Physical role functioning**	87.1 ± 28.9	100.0 (100.0-100.0)	86.8 ± 29.2	100.0 (100.0-100.0)	80.2 ± 34.6	100.0 (75.0-100.0)
**Emotional role functioning**	91.6 ± 24.2	100.0 (100.0-100.0)	90.8 ± 29.2	100.0 (100.0-100.0)	86.0 ± 30.7	100.0 (100.0-100.0)
**Mental health**	80.2 ± 13.3	84.0 (72.0-88.0)	79.8 ± 13.7	84.0 (72.0-88.0)	76.6 ± 16.2	80.0 (68.0-88.0)
**Vitality**	69.6 ± 16.7	70.0 (60.0-80.0)	67.9 ± 17.0	70.0 (60.0-80.0)	62.6 ± 18.9	65.0 (50.0-75.0)
**Bodily pain**	85.6 ± 18.2	89.8 (77.6-100.0)	84.5 ± 19.0	89.8 (67.3-100.0)	79.8 ± 22.6	89.8 (67.3-100.0)
**General health**	74.3 ± 16.4	75.0 (65.0-85.0)	72.0 ± 16.6	75.0 (65.0-85.0)	65.2 ± 19.0	65.0 (55.0-80.0)
	**Thrombocytopenia**		**Platelets in normal range**		**Thrombocytosis**	
	Mean ± SD	Median (IQR)	Mean ± SD	Median (IQR)	Mean ± SD	Median (IQR)
**Physical functioning**	90.4 ± 14.4	95.0 (90.0-100.0)	90.6 ± 14.3	95.0 (90.0-100.0)	86.8 ± 18.1	95.0 (85.0-100.0)
**Social functioning**	88.9 ± 17.3	100.0 (87.5-100.0)	87.6 ± 18.0	100.0 (75.0-100.0)	84.1 ± 21.1	87.5 (75.0-100.0)
**Physical role functioning**	86.2 ± 30.2	100.0 (100.0-100.0)	86.7 ± 29.3	100.0 (100.0-100.0)	81.4 ± 33.8	100.0 (75.0-100.0)
**Emotional role functioning**	92.0 ± 23.5	100.0 (100.0-100.0)	90.8 ± 25.3	100.0 (100.0-100.0)	88.3 ± 28.5	100.0 (100.0-100.0)
**Mental health**	81.7 ± 13.3	84.0 (72.0-92.0)	79.7 ± 13.8	84.0 (72.0-88.0)	78.0 ± 14.9	80.0 (72.0-88.0)
**Vitality**	70.0 ± 17.1	73.3 (60.0-83.8)	67.9 ± 17.0	70.0 (60.0-80.0)	64.3 ± 18.3	65.0 (50.0-75.0)
**Bodily pain**	86.0 ± 18.4	100.0 (77.6-100.0)	84.5 ± 19.1	89.8 (67.3-100.0)	80.1 ± 22.1	89.8 (67.3-100.0)
**General health**	71.8 ± 17.0	75.0 (60.0-85.0)	72.0 ± 16.7	75.0 (65.0-85.0)	68.3 ± 19.0	70.0 (60.0-80.0)
	**Neutropenia**		**Neutrophils in normal range**		**Neutrophilia**	
	Mean ± SD	Median (IQR)	Mean ± SD	Median (IQR)	Mean ± SD	Median (IQR)
**Physical functioning**	92.3 ± 12.6	95.0 (90.0-100.0)	90.6 ± 14.3	95.0 (90.0-100.0)	86.2 ± 18.7	95.0 (80.0-100.0)
**Social functioning**	88.6 ± 17.1	100.0 (87.5-100.0)	87.6 ± 18.0	100.0 (75.0-100.0)	83.4 ± 21.6	87.5 (75.0-100.0)
**Physical role functioning**	88.1 ± 27.8	100.0 (100.0-100.0)	86.7 ± 29.3	100.0 (100.0-100.0)	81.3 ± 34.2	100.0 (75.0-100.0)
**Emotional role functioning**	91.9 ± 23.8	100.0 (100.0-100.0)	90.8 ± 25.3	100.0 (100.0-100.0)	86.2 ± 30.3	100.0 (100.0-100.0)
**Mental health**	80.3 ± 13.1	84.0 (72.0-88.0)	79.8 ± 13.7	84.0 (72.0-88.0)	76.8 ± 16.0	80.0 (68.0-88.0)
**Vitality**	69.9 ± 16.3	70.0 (60.0-80.0)	67.9 ± 17.0	70.0 (60.0-80.0)	63.2 ± 18.6	65.0 (50.0-75.0)
**Bodily pain**	85.7 ± 18.1	89.8 (77.6-100.0)	84.5 ± 19.1	89.8 (67.3-100.0)	80.7 ± 22.4	89.8 (67.3-100.0)
**General health**	74.5 ± 16.0	75.0 (65.0-85.0)	72.0 ± 16.7	75.0 (65.0-85.0)	66.6 ± 19.1	70.0 (55.0-80.0)

	Lymphopenia		Lymphocytes in normal range		Lymphocytosis	
	Mean ± SD	Median (IQR)	Mean ± SD	Median (IQR)	Mean ± SD	Median (IQR)
**Physical functioning**	87.2 ± 18.3	95.0 (85.0-100.0)	90.7 ± 14.1	95.0 (90.0-100.0)	87.9 ± 16.8	95.0 (85.0-100.0)
**Social functioning**	84.5 ± 21.7	100.0 (75.0-100.0)	87.8 ± 17.9	100.0 (100.0-100.0)	84.9 ± 20.5	100.0 (75.0-100.0)
**Physical role functioning**	80.9 ± 34.9	100.0 (75.0-100.0)	86.8 ± 29.2	100.0 (100.0-100.0)	83.2 ± 32.5	100.0 (75.0-100.0)
**Emotional role functioning**	88.7 ± 28.3	100.0 (100.0-100.0)	90.9 ± 25.1	100.0 (100.0-100.0)	87.6 ± 29.1	100.0 (100.0-100.0)
**Mental health**	79.9 ± 13.4	84.0 (72.0-88.0)	79.8 ± 13.6	84.0 (72.0-88.0)	77.5 ± 15.5	80.0 (68.0-88.0)
**Vitality**	67.5 ± 18.9	70.0 (55.0-80.0)	68.1 ± 17.0	70.0 (60.0-80.0)	64.6 ± 18.3	65.0 (50.0-80.0)
**Bodily pain**	82.9 ± 21.3	89.8 (67.3-100.0)	84.6 ± 19.0	89.8 (67.3-100.0)	81.9 ± 21.1	89.8 (67.3-100.0)
**General health**	67.5 ± 21.3	75.0 (55.0-80.0)	72.2 ± 16.6	75.0 (65.0-85.0)	67.7 ± 18.2	70.0 (55.0-80.0)
	**Monocytopenia**		**Monocytes in normal range**		**Monocytosis**	
	Mean ± SD	Median (IQR)	Mean ± SD	Median (IQR)	Mean ± SD	Median (IQR)
**Physical functioning**	91.9 ± 13.0	95.0 (90.0-100.0)	90.6 ± 14.3	95.0 (90.0-100.0)	86.7 ± 18.4	95.0 (85.0-100.0)
**Social functioning**	87.5 ± 18.1	100.0 (75.0-100.0)	87.7 ± 18.0	100.0 (75.0-100.0)	85.8 ± 20.2	100.0 (75.0-100.0)
**Physical role functioning**	87.6 ± 28.4	100.0 (100.0-100.0)	86.6 ± 29.4	100.0 (100.0-100.0)	83.2 ± 32.4	100.0 (75.0-100.0)
**Emotional role functioning**	91.3 ± 25.0	100.0 (100.0-100.0)	90.8 ± 25.2	100.0 (100.0-100.0)	87.8 ± 28.9	100.0 (100.0-100.0)
**Mental health**	79.5 ± 13.4	84.0 (72.0-88.0)	80.0 ± 13.7	84.0 (72.0-88.0)	76.1 ± 15.5	84.0 (72.0-92.0)
**Vitality**	67.8 ± 16.7	70.0 (60.0-80.0)	67.9 ± 17.0	70.0 (60.0-80.0)	66.1 ± 18.3	70.0 (55.0-80.0)
**Bodily pain**	85.1 ± 18.3	89.8 (77.6-100.0)	84.4 ± 19.1	89.8 (67.3-100.0)	83.1 ± 20.9	89.8 (67.3-100.0)
**General health**	73.7 ± 16.3	75.0 (65.0-85.0)	71.9 ± 16.7	75.0 (65.0-85.0)	67.5 ± 18.1	70.0 (55.0-80.0)
	**NLR lowest quintile**		**NLR 2nd to 4th quintile**		**NLR highest quintile**	
	Mean ± SD	Median (IQR)	Mean ± SD	Median (IQR)	Mean ± SD	Median (IQR)
**Physical functioning**	91.6 ± 13.4	95.0 (90.0-100.0)	90.6 ± 14.3	95.0 (90.0-100.0)	89.1 ± 15.7	95.0 (85.0-100.0)
**Social functioning**	88.3 ± 17.6	100.0 (75.0-100.0)	87.5 ± 18.2	100.0 (75.0-100.0)	86.5 ± 18.8	100.0 (75.0-100.0)
**Physical role functioning**	87.9 ± 28.0	100.0 (100.0-100.0)	86.6 ± 29.4	100.0 (100.0-100.0)	84.7 ± 31.1	100.0 (100.0-100.0)
**Emotional role functioning**	91.3 ± 24.6	100.0 (100.0-100.0)	90.7 ± 25.4	100.0 (100.0-100.0)	89.6 ± 26.8	100.0 (100.0-100.0)
**Mental health**	79.8 ± 13.6	84.0 (72.0-88.0)	79.7 ± 13.8	84.0 (72.0-88.0)	79.2 ± 14.1	84.0 (72.0-88.0)
**Vitality**	68.8 ± 16.8	70.0 (60.0-80.0)	67.7 ± 17.1	70.0 (55.0-80.0)	66.8 ± 17.4	70.0 (55.0-80.0)
**Bodily pain**	85.1 ± 18.5	89.8 (77.6-100.0)	84.5 ± 19.2	89.8 (67.3-100.0)	83.3 ± 19.9	89.8 (67.3-100.0)
**General health**	72.9 ± 16.4	75.0 (65.0-85.0)	71.8 ± 16.7	75.0 (62.5-85.0)	70.7 ± 17.3	70.0 (60.0-85.0)

SDC, Table 2. Absolute scores for the different domains of the RAND-36 health survey, stratified according to differential blood cell count abnormality. NLR, neutrophil to lymphocyte ratio; WBC, white blood cell.

SDC, Figure 1. Distribution of white blood cell and platelet counts in the evaluable Lifelines cohort (n=143191). In order to improve visibility, figures were cropped at the extremes and a minor proportion of outliers in platelet count (>600 x 10^9/L, n=52) and white blood cell count (>20 x 10^9/L, n=34) were removed. WBC, white blood cell.

SDC, Figure 2. Distribution of differential white blood cell counts in the evaluable Lifelines cohort (n=140890). In order to improve visibility, figures were cropped at the extremes and a minor proportion of outliers in neutrophil count (>12 x 10^9/L, n=57), lymphocyte count (>5 x 10^9/L, n=3), monocyte count (>1.3 x 10^9/L, n=67), eosinophil count (>1.3 x 10^9/L, n=86), basophil count (>0.25 x 10^9/L, n=8) and neutrophil to lymphocyte ratio (>10, n=50) were removed. NLR, neutrophil to lymphocyte ratio.

SDC, Figure 3. Forest plots demonstrating the odds ratios for having a lower score than the (age- and sex-specific) 25th percentile cut-off per HRQoL subscale, according to different strata of blood cell counts. Logistic regression analyses included body mass index, smoking status, alcohol use, and number of medications, presence of anemia and mean corpuscular volume as covariates. The following ranges in peripheral blood cell counts were used as the reference group: white blood cell count 4-6 x 10^9/L; platelets 250-300 x 10^9/L; neutrophils 3-4 x 10^9/L; lymphocytes 1.6-2.0 x 10^9/L; monocytes 0.4-0.5 x 10^9/L; and NLR in the 3rd quintile. Circles indicate odds ratios for each domain, with horizontal lines corresponding to 95% confidence intervals. NLR, neutrophil to lymphocyte ratio; WBC, white blood cell.

SDC, Figure 4. Percentage of individuals with a score below the age- and sex-specific cut-off value for the different domains of the RAND-36 health survey, stratified according to differential blood cell count abnormality and age group (<60 years and ≥ 60 years). A circumflex (^) indicates a significant difference in the proportion below the age- and sex-specific cut-off value for individuals with neutropenia, lymphopenia, monocytopenia or lowest NLR quintile, as compared to individuals with a normal blood cell count. An asterisk (*) indicates a significant difference in the proportion below the age- and sex-specific cut-off value for individuals with neutrophilia, lymphocytosis, monocytosis, eosinophilia or highest NLR quintile, as compared to individuals with a normal blood cell count. BP, bodily pain; GH, general health; MH, mental health; NLR, neutrophil to lymphocyte ratio; PF, physical functioning; RE, emotional role functioning; RF, physical role functioning; SF, social functioning; VT, vitality; WBC, white blood cell.

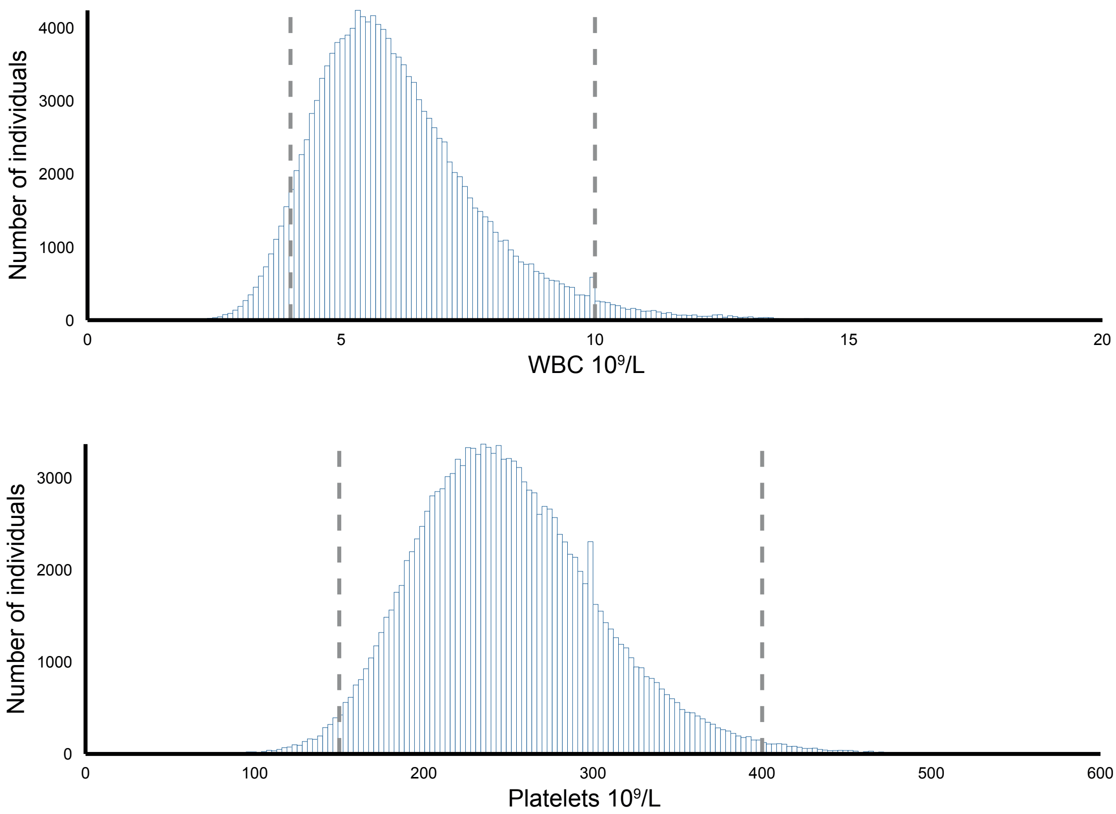
Number of individuals
4000
3000
2000
1000
0
0
5
10
15
20
WBC 10⁹/L
Number of individuals
3000
2000
1000
0
0
100
200
300
400
500
600
Platelets 10⁹/L

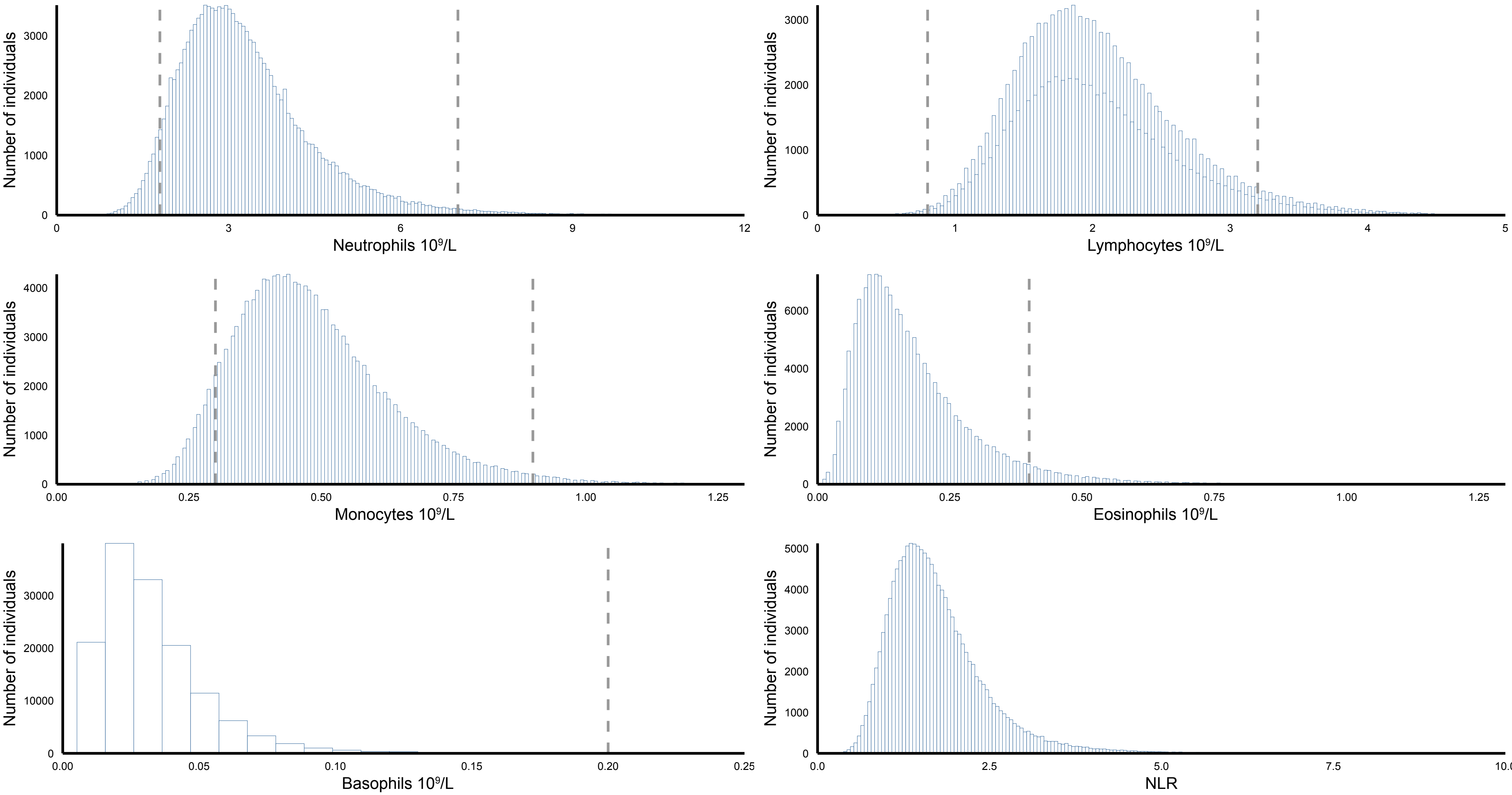
Number of individuals
Neutrophils 10^9/L
Number of individuals
Lymphocytes 10^9/L
Number of individuals
Monocytes 10^9/L
Number of individuals
Eosinophils 10^9/L
Number of individuals
Basophils 10^9/L
Number of individuals
NLR

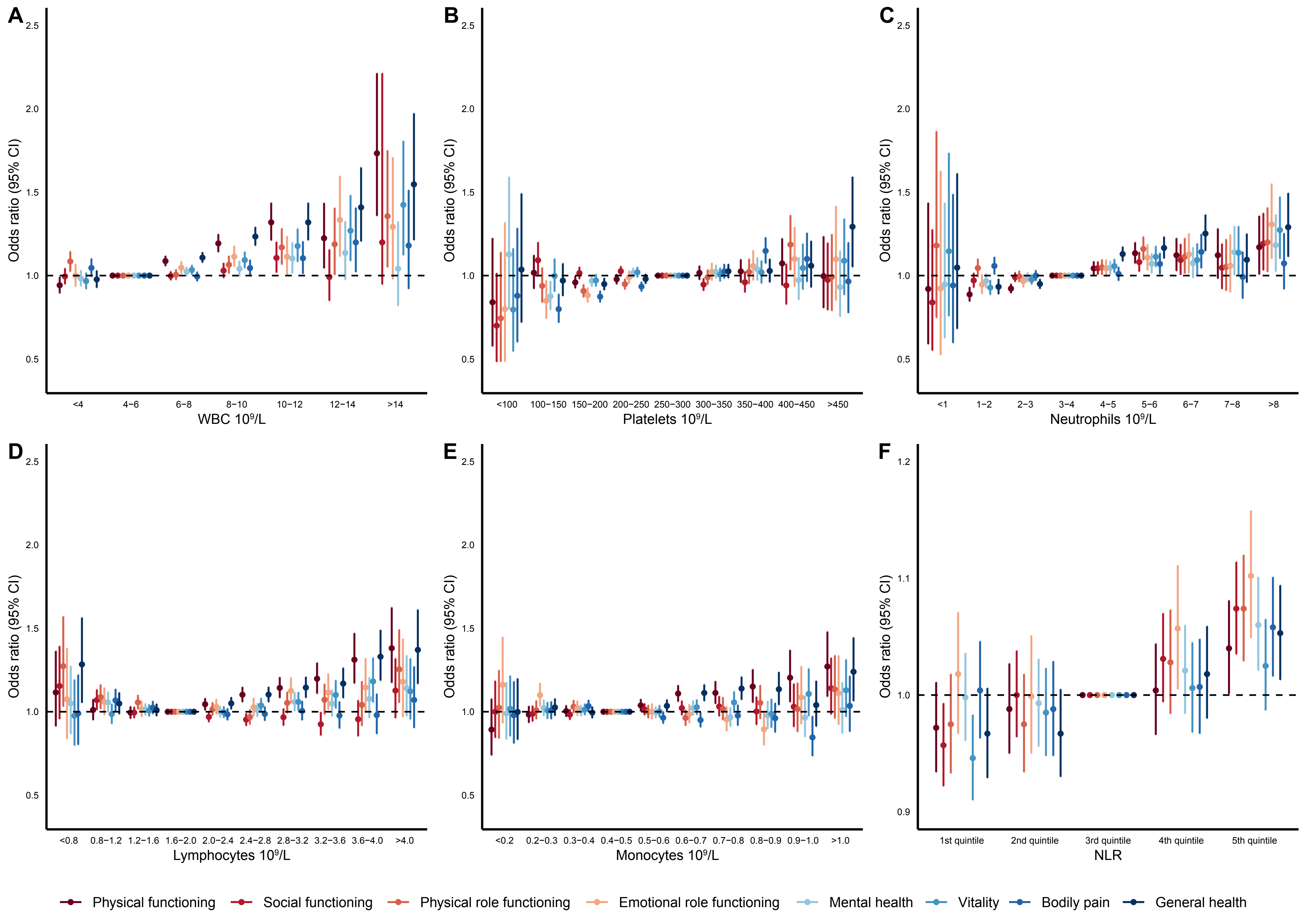
A
B
C
D
E
F
Odds ratio (95% CI)
WBC 10⁹/L
<4
4–6
6–8
8–10
10–12
12–14
>14
Platelets 10⁹/L
<100
100–150
150–200
200–250
250–300
300–350
350–400
400–450
>450
Neutrophils 10⁹/L
<1
1–2
2–3
3–4
4–5
5–6
6–7
7–8
>8
Lymphocytes 10⁹/L
<0.8
0.8–1.2
1.2–1.6
1.6–2.0
2.0–2.4
2.4–2.8
2.8–3.2
3.2–3.6
3.6–4.0
>4.0
Monocytes 10⁹/L
<0.2
0.2–0.3
0.3–0.4
0.4–0.5
0.5–0.6
0.6–0.7
0.7–0.8
0.8–0.9
0.9–1.0
>1.0
NLR
1st quintile
2nd quintile
3rd quintile
4th quintile
5th quintile
Physical functioning
Social functioning
Physical role functioning
Emotional role functioning
Mental health
Vitality
Bodily pain
General health

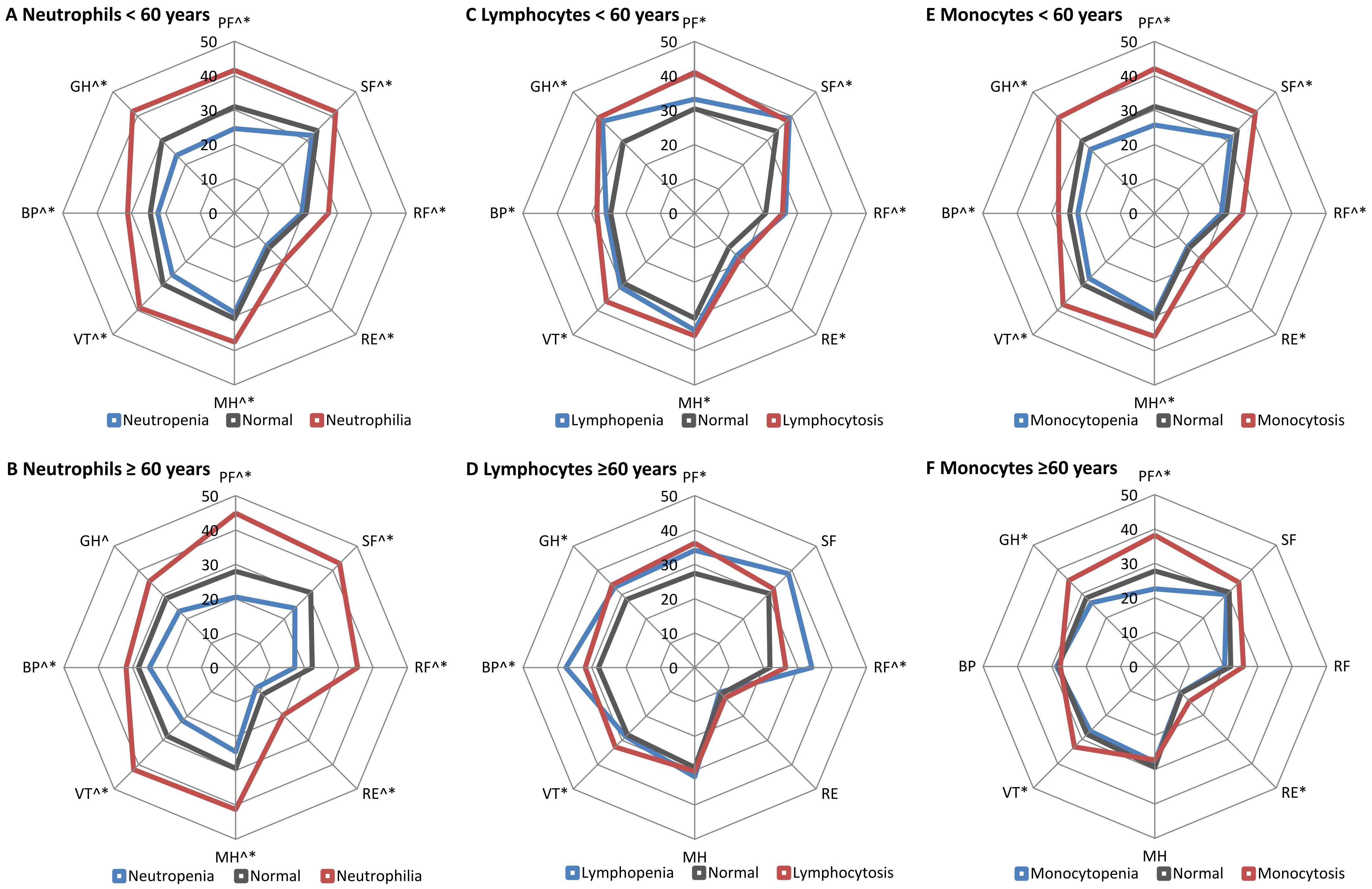
A Neutrophils < 60 years
PF^*
SF^*
RF^*
RE^*
MH^*
VT^*
BP^*
GH^*
0
10
20
30
40
50
Neutropenia
Normal
Neutrophilia
C Lymphocytes < 60 years
PF*
SF^*
RF^*
RE*
MH*
VT*
BP*
GH^*
Lymphopenia
Normal
Lymphocytosis
E Monocytes < 60 years
PF^*
SF^*
RF^*
RE*
MH^*
VT^*
BP^*
GH^*
Monocytopenia
Normal
Monocytosis
B Neutrophils ≥ 60 years
PF^*
SF^*
RF^*
RE^*
MH^*
VT^*
BP^*
GH^
Neutropenia
Normal
Neutrophilia
D Lymphocytes ≥60 years
PF*
SF
RF^*
RE
MH
VT*
BP^*
GH*
Lymphopenia
Normal
Lymphocytosis
F Monocytes ≥60 years
PF^*
SF
RF
RE*
MH
VT*
BP
GH*
Monocytopenia
Normal
Monocytosis

G Eosinophils < 60 years

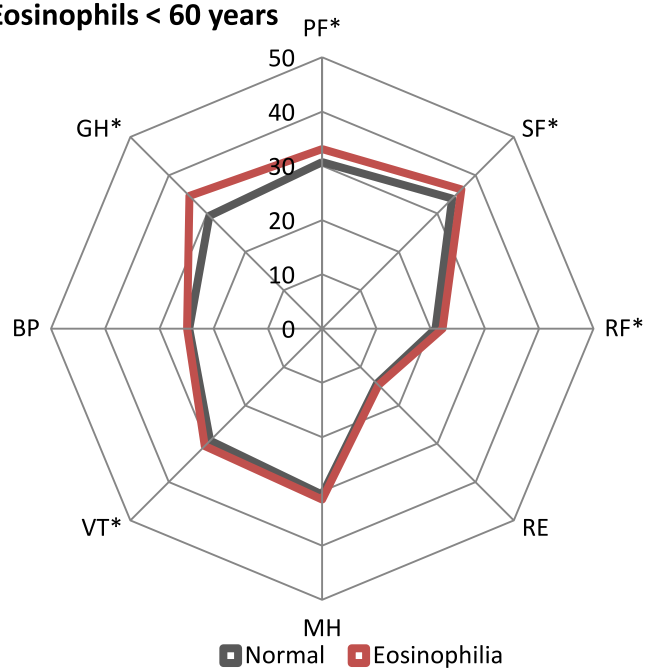

H Eosinophils ≥ 60 years

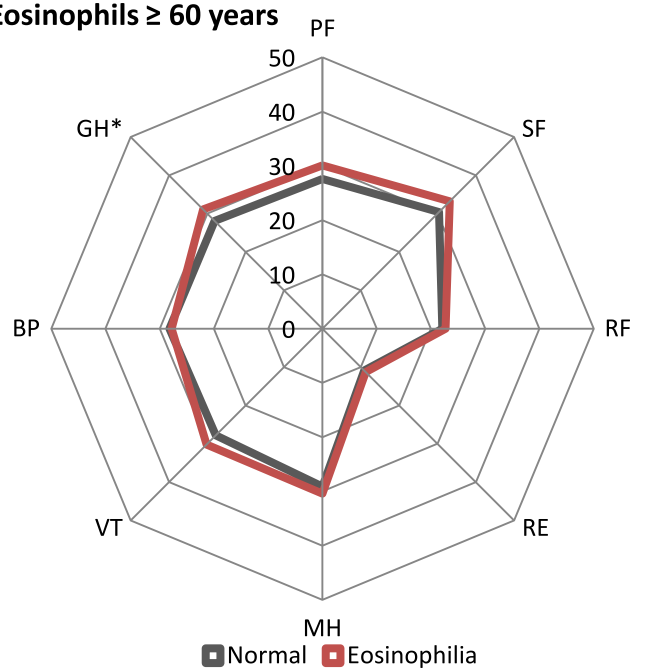

I NLR < 60 years

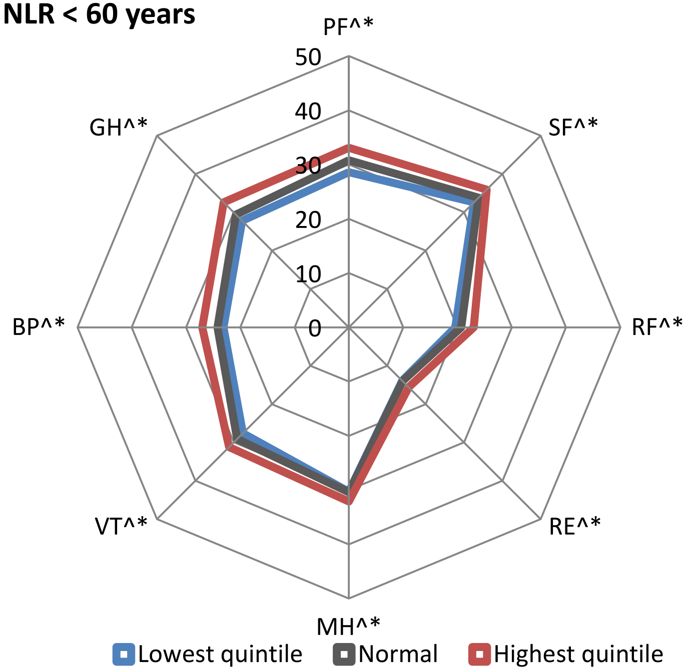

J NLR ≥ 60 years

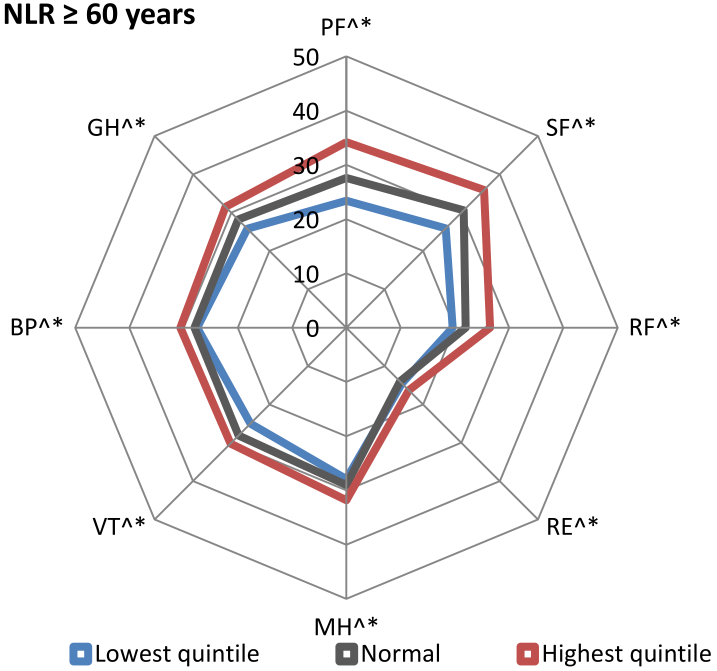

Supplement: Supplementary file 1 [file hs9-5-e512-s001.pdf]
